# Relationships Among Type D Personality, Symptoms, Depression and Anxiety, and Quality of Life in Patients With Gastroesophageal Reflux Disease

**DOI:** 10.1097/jnr.0000000000000752

**Published:** 2026-06-17

**Authors:** Yusun PARK, Boram CHA, Hye Young KIM, Sung Reul KIM

**Affiliations:** 1 Department of Nursing, The University of Suwon, Hwaseong, Gyeonggi-do, South Korea; 2 Department of Internal Medicine, Inha University Hospital, Inha University School of Medicine, Incheon, South Korea; 3 College of Nursing, Jeonbuk Research Institute of Nursing Science, Jeonbuk National University, Jeonju, South Korea; 4 College of Nursing, Korea University Nursing Research Institute, Korea University, Seoul, South Korea; ^†^Contributed equally

**Keywords:** depression, gastroesophageal reflux, quality of life, symptom, Type D personality

## Abstract

**Background:**

Patients with gastroesophageal reflux disease (GERD) often experience typical or atypical symptoms accompanied by depression, anxiety, and low quality of life (QoL). In patients with various diseases, having a Type D personality has been linked to an elevated risk of developing symptoms such as depression and anxiety, as well as reduced QoL.

**Purpose:**

The objectives of this study were to examine the influence of Type D personality on GERD symptoms, depression and anxiety, and QoL and to identify the causal relationships among the variables influencing QoL in patients with GERD.

**Methods:**

A cross-sectional correlational design and convenience sampling were used, and 149 patients with GERD were enrolled as study participants from the gastroenterology outpatient clinic of a university hospital in the Republic of Korea. Data were collected using structured questionnaires and tested using SPSS 28.0 and Amos 23.0.

**Results:**

Approximately 46% of the participants met the criteria for inclusion in the Type D personality group. This group exhibited higher levels of depression and anxiety and diminished QoL than the non–Type D personality group. In the multiple regression model, GERD symptoms, depression and Type D personality were found to have a significant influence on QoL. In the structural equation model, GERD symptoms, depression, and Type D personality were shown to affect QoL, explaining 51.7% of the total variance in QoL. Two factors, namely depression and GERD symptoms, were identified as mediators of the association between Type D personality and QoL.

**Conclusions:**

The results of this study indicate a relatively high frequency of Type D personality among patients with GERD. When assessing QoL in patients with GERD, it is essential to consider the influence of Type D personality in conjunction with symptom experience and the presence of depression and anxiety. In particular, managing depression and symptoms in patients with GERD and a Type D personality may offer an effective approach to improving QoL in this patient group.

## Introduction

The global prevalence of gastroesophageal reflux disease (GERD) is 13.98%, with an estimated 1.03 billion sufferers worldwide (Nirwan et al., 2020). A large community-based survey of 71,000 individuals in the United States revealed 44.1% exhibited symptoms of GERD and that 35.1% had received related treatments (Delshad et al., 2020). GERD is characterized by the reflux of stomach contents into the esophagus or beyond, reaching the oral cavity, larynx, or lungs, and leading to symptoms or complications (Radovanovic et al., 2021). If left untreated, GERD can cause serious complications, including esophageal strictures, esophagitis, Barrett’s esophagus, and esophageal adenocarcinoma (Maret-Ouda et al., 2020). GERD has also been linked to chronic respiratory diseases such as aspiration bronchitis and chronic obstructive pulmonary disease (Lee et al., 2020).

Patients with GERD typically experience symptoms such as heartburn, retrosternal pain, and regurgitation, along with atypical symptoms such as a sensation of having a lump in the throat (globus), sore throat, hoarseness, or chronic cough (Jung et al., 2018). These symptoms have been shown to negatively affect quality of life (QoL) in these patients (Alzahrani et al., 2023; Fuchs et al., 2022), with approximately 83.3% of patients with GERD reporting a poor QoL (Alzahrani et al., 2023). Therefore, GERD-related symptoms and QoL are pivotal factors in the treatment and management of patients with GERD (Chen & Brady, 2019).

Psychological factors, including depression, anxiety, and stress, are widely acknowledged as significant risk factors for symptoms and QoL in GERD patients (Wickramasinghe et al., 2023; Zamani et al., 2023). Stress can precede conditions such as functional dyspepsia and heartburn and can aggravate GERD symptoms by amplifying perceptual responses to intraesophageal acid stimuli via central mechanisms without increasing the volume of acid reflux (Sandhu & Fass, 2018). Stress can increase the perception of intraesophageal acid exposure and impact health behaviors such as alcohol consumption, diet, physical activity, and smoking, all of which affect the risk of reflux (Boulton & Dettmar, 2022). Moreover, stress may diminish the effectiveness of treatment in GERD patients (Sandhu & Fass, 2018). Stress-relieving interventions have previously been shown to enhance the QoL and symptom experience of patients with GERD (Chandran et al., 2023).

Type D personality, associated with tendencies toward negative affectivity and social inhibition, has been strongly associated with stress (Denollet, 2005). Patients with chronic diseases frequently experience depression and anxiety (Fattouh et al., 2019), and social isolation or withdrawal is closely related to their physical and psychosocial symptoms experience (Iovino et al., 2023; Ahn et al., 2022). The findings of prior research show people with Type D personality to be more prone to chronic stress (Gębska et al., 2022) and tend to have worse disease outcomes (Raykh et al., 2022). Patients with a Type D personality and a chronic illness, such as psoriasis, stroke, diabetes, or brain tumor, have been shown to experience higher depression and anxiety, more severe symptoms, reduced self-management, and lower QoL than their non–Type D personality peers with these illnesses (Aguayo Carreras et al., 2020; Akbari et al., 2020; Kim, Kim, et al., 2021; Kim, Nho, et al., 2021).

Patients with GERD frequently experience depression and anxiety, and GERD symptoms that affect their social functioning (Mohammad et al., 2019). Moreover, having a Type D personality has been related to unhealthy behaviors (Buczkowska et al., 2022), which are significant contributors to the onset and progression of symptoms in patients with GERD (Wang et al., 2021). Specifically, patients with functional gastrointestinal disorders report impaired health-related QoL and severe gastrointestinal symptoms (Hansel et al., 2010). Given the relationship between Type D personality and higher depression, anxiety, and stress sensitivity, this personality type may be significantly related to depression and anxiety, GERD symptoms, and QoL in patients diagnosed with GERD. The focus of most previous related studies has been on investigating the associations between Type D personality, symptoms, and QoL in patients with chronic diseases, with few studies reporting a causal relationship between the direct and indirect effects of Type D personality on symptoms and QoL. Moreover, few studies have been published on the influence of Type D personality on symptoms, depression, anxiety, or QoL in patients with GERD. Therefore, this study was designed to examine the influence of Type D personality on GERD symptoms, depression, anxiety, and QoL and to identify the causal relationships among the variables influencing QoL in patients with GERD.

## Methods

### Sample

In this descriptive, cross-sectional study, patients were recruited using convenience sampling from the gastroenterology outpatient clinic of a university hospital in the Republic of Korea. The inclusion criteria were the following: (a) diagnosis of GERD by a gastroenterologist, (b) aged 20 years or older, and (c) able to communicate in Korean. The exclusion criteria were having a chronic medical condition such as chronic renal failure, heart failure, or active cancer that may affect symptoms, health behaviors, and QoL.

Multiple linear regression was performed to determine the appropriate sample size using G*Power 3.1.9.7. The sample size estimation was based on a significance level of .05, power of 0.80, median effect size of 0.15, regression analysis (Faul et al., 2009), and 17 predictors. Based on these criteria, a minimum sample size of 146 was required. In addition, the suggestion of Kline (2023) that a sample size within the range of 100–200 is generally needed for structural equation modeling (SEM) to ensure sufficient statistical power and robustness was followed.

### Data Collection

Data were collected between July 2022 and March 2023 using a structured questionnaire.

#### Type D Personality

Type D personality was assessed using the Korean version of the Type D Personality Scale-14 (Denollet, 2005; Lim et al., 2011). This scale consists of 14 items distributed across two domains: negative affectivity and social inhibition. Each item is evaluated using a 5-point Likert scale ranging from 0 to 4, with respondents earning subtotal scores of 10 or above in both domains classified as having a Type D personality. In this study, the Cronbach’s alpha was .86 for negative affectivity and .84 for social inhibition.

#### GERD Symptoms

The GERD symptoms experience of participants was evaluated using the Korean version of the Self-Evaluation Questionnaire for Gastroesophageal Reflux Disease (SEQ-GERD; Jung et al., 2018). This scale has been validated for reliability, including internal consistency (Cronbach’s alpha=.60–.94), test–retest reliability (intraclass correlation coefficient=.67–.95), and validity through its relationship to QoL (*r*=−.54) in the Korean context (Jung et al., 2018). The scale comprises six items distributed across two dimensions, namely the domains of GERD and upper GI symptoms. Each item is designed to evaluate symptom severity and frequency using a 5-point Likert scale, with scores ranging from 1 to 5. The scores are aggregated to create a sum score for the frequency and severity of each symptom, with a total score ranging from 12 to 60, and higher scores indicating a stronger symptom experience. The Cronbach’s alpha for this study was .79.

#### Depression

The Korean version of the Patient Health Questionnaire-9 was used to assess depression in this study (Han et al., 2008; Kroenke et al., 2001). The scale comprises nine items, each of which reflects a single criterion for major depressive disorder. Each item is rated on a 4-point Likert scale, with scores ranging from 0 to 3. Total scores range from 0 to 27, with higher scores indicating more severe depressive symptoms. The Cronbach’s alpha for this study was .88.

#### Anxiety

The Korean version of the Generalized Anxiety Disorder-7 was used to assess anxiety in this study (Seo & Park, 2015; Spitzer et al., 2006). The Generalized Anxiety Disorder-7 consists of seven items, each addressing a core symptom of generalized anxiety disorder. Each item is rated on a 4-point Likert scale, with scores ranging from 0 to 3. Total scale scores range from 0 to 21, with higher scores indicating more severe anxiety symptoms. The Cronbach’s alpha for this study was .90.

#### Quality of Life

QoL was evaluated using the Korean version of the Quality of Life Questionnaire for Gastroesophageal Reflux Disease (Chan et al., 2010; Jung et al., 2018), which comprises 17 items distributed across four dimensions: daily activity (DA), treatment effect (TE), diet (DI), and psychological well-being (PW). Each item is rated on a 5-point Likert scale, with scores ranging from 0 to 4. Total scale scores range from 0 to 100, with higher scores indicating higher (better) QoL. Confirmatory factor analysis (CFA) was conducted to test the construct validity of this scale, with factor loadings from .674 to .900 and critical ratios (CR) ranging from 9.117 to 12.798 (*p*<.001). The model was shown to be suitable, with normed χ²=2.313 (*p*=.128), goodness of fit index (GFI)=.992, comparative fit index (CFI)=.996, normed fit index (NFI)=.993, Tucker-Lewis index (TLI)=.977, and standardized root mean residual (SRMR)=.010. The Cronbach’s alpha for this scale in this study was .86 for DA, .84 for TE, .86 for DI, and .90 for PW.

#### General and Clinical Characteristics and Health Behaviors

Participant demographic characteristics, including age, gender, educational level, marital status, family income, and occupation, were collected using a self-report datasheet and evaluated. Clinical characteristics were evaluated using interviews and medical records, and disease duration was also assessed. The health behaviors of the participants were evaluated using the following seven items derived from Zhang et al. (2021): (a) body mass index, (b) alcohol consumption, (c) tea and coffee intake, (d) eating habits (late-night snacking, dinner within 2 hours of going to bed, frequency of breakfast, and quick eating), (e) exercise, (f) insufficient sleep (subjective perception of insufficient sleep), and (g) cigarette use. The researcher measured body mass index, and the remaining items were investigated using a questionnaire.

### Data Analysis

Statistical analyses were conducted using IBM SPSS Statistics Version 28.0 (IBM Corp., Armonk, NY, USA) and AMOS 23.0. The demographic and clinical characteristics and study variables were analyzed using descriptive statistics. Type D personality was evaluated in relation to demographic and clinical characteristics; symptoms, depression and anxiety, health behaviors, and QoL were evaluated using the independent *t* test, χ^2^ test, Mann–Whitney *U* test, and Fisher’s exact test; QoL scores were analyzed in relation to demographic and clinical characteristics and health behaviors using one-way analysis of variance, independent *t* tests, and post hoc Scheffe tests; and Pearson’s correlation coefficients were used to assess the correlations among the study variables. Stepwise multiple regression analysis was conducted to elucidate the factors of influence on QoL.

In addition, an SEM analysis was conducted to test the causal relationship between the variables affecting QoL. The skewness and kurtosis were tested for normality. Tolerance, variance inflation factor (VIF), and Pearson’s correlation coefficient were evaluated for multicollinearity. To test the fit of the data to the hypothetical model, χ² (*p*), normed χ², the GFI, the CFI, the NFI, incremental fit index (IFI), and standardized root mean residual (SRMR) were performed. The squared multiple correlations (SMC) of the study variables with QoL in the hypothetical model were also evaluated. Bootstrapping was employed to analyze the indirect, direct, and total effects. The indirect effect of each variable on the multimediation model with multiple indirect effects was tested using a phantom variable. Two-tailed *p* < .05 was considered to indicate statistical significance.

### Ethical Considerations

This study was approved by the institutional review board of Inha University Hospital (No. 2021-04-054). All of the participants were informed that they could withdraw from the study at any time, and their informed written consent was obtained.

## Results

### Participant Demographic and Clinical Characteristics and Health Behaviors

Of the 177 returned questionnaires, 24 were excluded because of incomplete responses or over 50% of items left unanswered. Thus, data from 149 participants were included in the final analysis.

Participant demographic and clinical characteristics and health behavior information are summarized in Table 1. The average age of the participants was 55.75 (*SD*=13.96) years, 84 (57.1%) were women, 51 (34.2%) had a disease duration of 36 months or more, 48% exercised for more than 30 min per day, and 66.2% reported insufficient sleep.

**Table 1:** Between-Group Comparisons of Demographic and Clinical Characteristics, Health Behaviors, GERD Symptoms, Depression and Anxiety, and Quality of Life (N = 149)

Variable	*n* (%)				
	Total (*n*=149)	Type D Group (*n*=69)	Non–Type D Group (*n*=80)	*t* or χ^2^	*p*
Age (years; *M* and *SD*)	55.75±13.96	54.63±13.88	56.71±14.04	−0.90	.370
Gender ^a^				0.51	.507
Men	63 (42.9)	27 (39.7)	36 (45.6)		
Women	84 (57.1)	41 (60.3)	43 (54.4)		
Marital status ^a^				0.71	.480
Married	94 (65.7)	41 (62.1)	53 (68.8)		
Not married	49 (34.3)	25 (37.9)	24 (31.2)		
Educational level ^a^				0.04	.867
≤ High school	58 (39.7)	26 (38.8)	32 (40.5)		
≥ College	88 (60.3)	41 (61.2)	47 (59.5)		
Monthly household income (10,000 KRW/month) ^a^				0.45	.501
< 300	34 (24.3)	17 (27.0)	17 (22.1)		
≥ 300	106 (75.7)	46 (73.0)	60 (77.9)		
Occupation ^a^				0.83	.404
No	67 (46.5)	28 (42.4)	39 (50.0)		
Yes	77 (53.5)	38 (57.6)	39 (50.0)		
Duration of disease (month)				0.23	.632
< 36	98 (65.8)	44 (63.8)	54 (67.5)		
≥ 36	51 (34.2)	25 (36.2)	26 (32.5)		
Body mass index (kg/m^2^; *M* and *SD*) ^a^	23.01±3.55	22.88±3.68	23.13±3.45	−0.41	.683
Classification of body mass index (kg/m^2^) ^a^				2.28	.517
< 18.5	10 (7.1)	6 (9.1)	4 (5.3)		
18.5–22.9	62 (44.3)	31 (47.0)	31 (40.8)		
23.0–24.9	35 (25.0)	13 (19.7)	22 (28.9)		
≥ 25	35 (25.0)	16 (24.2)	19 (25.0)		
Alcohol consumption				2.22	.155
No	104 (69.8)	44 (63.8)	60 (75.0)		
Yes	45 (30.2)	25 (36.2)	20 (25.0)		
Tea and coffee (times)				2.75	.253
Hardly drinking	58 (38.9)	22 (26.9)	36 (45.0)		
≤ 6 times/week	38 (25.5)	19 (27.5)	19 (23.8)		
≥ daily	53 (35.6)	28 (40.6)	25 (31.3)		
Eating habits					
Frequency of late-night snacking (times/week)				3.49	.076
≥ 3	33 (22.1)	20 (29.0)	13 (16.3)		
<3	116 (77.9)	49 (71.0)	67 (83.7)		
Dinner within 2 hr of going to bed				3.64	.066
No	90 (60.4)	36 (52.2)	54 (67.5)		
Yes	59 (39.6)	33 (47.8)	26 (32.5)		
Frequency of breakfast (time/week)				2.92	.102
≥ 3	76 (51.0)	30 (43.5)	46 (57.5)		
<3	73 (49.0)	39 (56.5)	34 (42.5)		
Quickly eating				0.68	.510
No	68 (45.6)	29 (42.6)	39 (48.8)		
Yes	81 (54.4)	40 (58.0)	41 (51.2)		
Average exercise (min/day) ^a^				2.83	.102
<30	77 (52.0)	41 (59.4)	36 (45.6)		
≥ 30	71 (48.0)	28 (40.6)	43 (54.4)		
Insufficient sleep ^a^				5.91	.023
No	50 (33.8)	16 (23.5)	34 (42.5)		
Yes	98 (66.2)	52 (76.5)	46 (57.5)		
Cigarette use				0.13	.811
No	129 (86.6)	59 (85.5)	70 (87.5)		
Yes	20 (13.4)	10 (14.5)	10 (12.5)		

	***M*±*SD***	***M*±*SD***	***M*±*SD***	***t***	***p***
GERD symptoms	24.28±8.56	25.62±8.20	23.13±8.74	1.79	.076
Depression	5.61±5.21	7.87±5.78	3.66±3.73	5.20	<.001
Anxiety	4.36±4.39	6.45±4.73	2.56±3.13	5.99	<.001
Quality of life					
Total	52.80±23.86	44.48±19.97	59.98±24.71	−4.23	<.001
Daily activity	66.56±22.40	59.67±21.28	72.50±21.74	−3.63	<.001
Treatment effect	55.17±28.41	44.98±24.19	63.96±28.98	−4.36	<.001
Diet	50.97±29.39	43.03±26.54	57.81±30.16	−3.15	<.001
Psychological well-being	38.87±30.35	30.25±25.31	46.30±32.45	−3.39	<.001

*Note.* GERD = gastroesophageal reflux disease; KRW = South Korean Won.

^a^ These data contain missing data.

### Between-Group Comparisons of Demographic and Clinical Characteristics, Health Behaviors, GERD symptoms, Depression and Anxiety, and QoL

The results of between-group comparisons of demographic and clinical characteristics, health behaviors, GERD symptoms, depression and anxiety, and QoL are shown in Table 1.

Sixty-nine (46.3%) of the participants were identified as having a Type D personality and assigned to the Type D personality group. Demographic and clinical characteristics did not differ significantly between the two groups. In terms of health behaviors, all variables were statistically similar between the groups with the exception of “frequency of insufficient sleep,” which was higher in the Type D personality group than the non–Type D personality group (χ^2^=5.91, *p*=.023).

The depression and anxiety scores of the Type D personality group were significantly higher than those of the personality non–Type D group (*t*=5.20, *p*<.001; *t*=5.99, *p*<.001, respectively). The GERD symptom score of the Type D personality group was higher than that of the personality non–Type D group; however, this difference was not statistically significant. In addition, the scores for total DA, TE, DI, and PW were significantly lower in the Type D personality group than in the personality non–Type D group (*t*=−4.23, *p*<.001; *t*=−3.63, *p*<.001; *t*=−4.36, *p*<.001; *t*=−3.15, *p*<.001; and *t*=−3.39, *p*<.001, respectively; Table 1).

### Factors Influencing QoL in Patients With GERD

The results for QoL scores categorized by demographic, clinical characteristic, and health behavior factors are summarized in Table 2. The QoL scores of participants with a disease duration of <3 years were higher than those with a disease duration of ≥3 years (*t*=−2.55, *p*= .012). Also, the QoL scores of participants with sufficient sleep were higher than those with insufficient sleep (*t*=2.30, *p*=.023). In the correlation analysis between QoL and the study variables, total QoL score was shown to correlate significantly with GERD symptoms (*r*=−.545, *p*<.001), depression (*r*=−.525, *p* <.001), and anxiety (*r*=−.462, *p* <.001). To identify the factors influencing QoL, we performed stepwise multiple regression using statistically significant independent variables (Table 2 and Supplementary Table 1, Supplemental Digital Content 1, http://links.lww.com/JNR/A10). The most significant factor influencing QoL was GERD symptoms (adjusted *R* ^2^=.292, *p* < .001), followed by depression (adjusted *R* ^2^=.106, *p* < .001) and Type D personality (adjusted *R* ^2^=.014, *p* < .001; Table 3).

**Table 2:** Quality of Life by Demographic, Clinical, and Health Behavior Characteristics (N= 149)

Variable	Mean±*SD*	*t* or *F* (*p*)
Gender		−1.61 (.110)
Male	56.18±25.05	
Female	49.87±22.36	
Marital status		−0.29 (.769)
Married	52.91±24.42	
Not married	51.69±21.98	
Educational level		−0.39 (.697)
≤ High school	53.73±23.92	
≥ College	52.17±23.52	
Monthly household income (10,000 won/month)		1.00 (.318)
<300	55.78±24.97	
≥300	51.09±23.32	
Occupation		−0.99 (.322)
No	50.16±21.75	
Yes	54.02±24.52	
Duration of disease (month)		−2.55 (.012)
<36	56.33±24.58	
≥36	46.03±21.01	
Body mass index (kg/m^2^)		4.31 ^a^ (.230)
<18.5	42.55±17.14	
18.5–22.9	51.76±23.61	
23.0–24.9	58.51±24.69	
≥25	48.44±22.04	
Alcohol consumption		−1.67 (.098)
No	50.67±23.44	
Yes	57.72±24.35	
Tea and coffee		2.28 (.106)
Hardly drinking	47.79±21.74	
≤ 6 times/week	54.45±26.86	
≥ daily	57.11±23.24	
Eating habits		
Frequency of late-night snack (times/week)		0.90 (.370)
≥3	49.50±21.49	
<3	53.74±24.44	
Dinner within 2 hr of going to bed		0.47 (.636)
No	53.56±25.18	
Yes	51.66±21.85	
Frequency of breakfast (times/week)		0.13 (.899)
<3	52.55±22.81	
≥3	53.05±24.98	
Quickly eating		0.49 (.628)
No	53.84±24.11	
Yes	51.93±23.76	
Average daily exercise (min)		0.43 (.670)
<30	52.07±24.56	
≥30	53.75±23.35	
Insufficient sleep		2.30 (.023)
No	59.14±24.82	
Yes	49.71±22.92	
Cigarette use		−0.02 ^b^ (.984)
No	53.84±24.11	
Yes	51.93±23.76	

^a^ Kruskal–Wallis test. ^b^ Mann–Whitney test.

**Table 3:** Stepwise Multiple Regression Analysis of Factors Influencing Quality of Life in Patients With GERD

Variable	Step 1			Step 2			Step 3		
	*B*	β	*t* (*p*)	*B*	β	*t* (*p*)	*B*	β	*t* (*p*)
(Constant)	90.056		17.97 (<.001)	89.136		19.27 (<.001)	91.181		19.51 (<.001)
GERD symptoms	−1.538	−.545	−7.86 (<.001)	−1.115	−.395	−5.62 (<.001)	−1.132	−.401	−5.77 (<.001)
Depression				−1.655	−.362	−5.15 (<.001)	−1.371	−.300	−3.98 (<.001)
Type D personality							−7.028	−.147	−2.12 (.036)
Adjusted *R* ^*2*^	.292			.398			.412		
Change in adjusted *R* ^*2*^				.106			.014		
*F*	61.73			49.51			35.30		
*p*	<.001			<.001			<.001		

*Note.* GERD = gastroesophageal reflux disease.

### GERD Symptoms and Depression as Mediators in the Relationship Between Type D Personality and QoL

An SEM analysis was conducted to confirm the causal relationships among the variables influencing QoL. The range of skewness was 0.150 to 1.449, and the range of kurtosis was −0.766 to 2.480. Thus, the assumption of the normality of the study variables was met for these criteria. The tolerance of the study variables was 0.712–0.836, and the VIF was 1.196–1.404. The correlation coefficients ranged from .408 to .741. These results indicate an acceptable level of multicollinearity between the observed variables. The hypothetical model was suitable; χ²=4.31 (*p* < .001), normed χ²=0.00, GFI=.922, CFI=.928, NFI=.909, IFI=.929, SRMR=.045. One of the six paths in the final path model was not supported (Type D personality → GERD symptoms).

The standardized estimates of the direct, indirect, and total effects of the exogenous variables on the endogenous variables are presented in Table 4. Type D personality was shown to have significant direct and total effects on depression (β=.404, 95% CI [.273, .509]; β=.404, 95% CI [.273, .509]) and a significant indirect effect on GERD symptoms (β=.169, 95% CI [.093, .266]), while depression was shown to have significant direct and total effects on GERD symptom (β=.417, 95% CI [.257, .578]; β=.417, 95% CI [.257, .578]). Type D personality (β=−.133, 95% CI [−.272, −.003); β=−.201, 95% CI [−.300, −.093]; β=−.334, 95% CI [−.473, −.184]) and depression (β=−.329, 95% CI [−.469, −.162]; β=−.193, 95% CI [−.301, −.118]; β=−.522, 95% CI [−.637, −.385]) were shown to have significant direct, indirect, and total effects on QoL, while GERD symptoms was shown to have significant direct and total effects on QoL (β=−.462, 95% CI [−.576, −.311]; β=−.462, 95% CI [−.576, −.311]). These findings indicate GERD symptoms and depression mediate the relationship between having a Type D personality and QoL.

**Table 4:** Standardized Direct, Indirect, and Total Effects of the Structural Equation Model

Endogenous Variable	Standardized Direct Effect		Standardized Indirect Effect		Standardized Total Effect		SMC
	β (*p*)	95% CI	β (*p*)	95% CI	β (*p*)	95% CI	
Depression							.163
Type D personality	.404 (.001)	[.273, .509]			.404 (.001)	[.273, .509]	
GERD symptoms							.167
Type D personality	−.023 (.774)	[−.163, .120]	.169 (.001)	[.093, .266]	.146 (.086)	[−.020, .297]	
Depression	.417 (.001)	[.257, .578]			.417 (.001)	[.257, .578]	
Quality of life							.517
Type D personality	−.133 (.047)	[−.272, −.003]	−.201 (.001)	[−.300, −.093]	−.334 (.001)	[−.473, −.184]	
Depression	−.329 (.001)	[−.469, −.162]	−.193 (.001)	[−.301, −.118]	−.522 (.001)	[−.637, −.385]	
GERD symptom	−.462 (.002)	[−.576, −.311]			−.462 (.002)	[−.576, −.311]	

*Note.* CI = confidence intervals; GERD = gastroesophageal reflux disease; SMC = squared multiple correlation.

**Figure 1:**
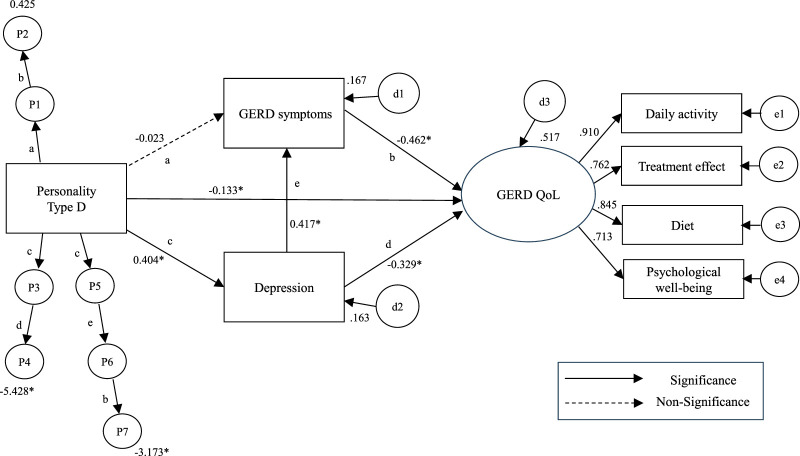
Path of the Hypothetical Model *Note* GERD = gastroesophageal reflux disease; QoL = quality of life. *p < .05.

To test specific indirect effects, phantom variables were employed for multiple indirect effects. The pathway of Type D personality → GERD symptoms → QoL was denoted as the phantom variable “P1” → “P2”; Type D personality → depression → QoL was denoted as “P3” → “P4”; and Type D personality → depression → GERD symptoms → QoL was denoted as “P5” → “P6” → “P7.” Two indirect effects were found to be significant, and one was found not to reach significance. The indirect effect of P2 (path of Type D personality → GERD symptoms → QoL) was *B*=0.425 (*p*=.774, 95% CI [−2.281, 3.306]); the indirect effect of P4 (path of Type D personality → depression → QoL) was *B*=−5.428 (*p*=.001, 95% CI [−2.571, −9.211]); and the indirect effect of P7 (path of Type D personality → depression → GERD symptoms → QoL) was *B*=−3.173 (*p*=.001, 95% CI [−1.633, −5.617]; Figure 1). The SMC values indicate Type D personality, depression, and GERD symptoms collectively explained 51.7% of the variance in QoL (Table 4 and Figure 1).

## Discussion

The strengths of this study are that it revealed the influence of Type D personality on depression, anxiety, GERD symptoms, and QoL in patients with GERD and a causal relationship between Type D personality and the variables affecting QoL. Thus, depression and GERD symptoms were shown to be mediators in the relationship between Type D personality and QoL. Although the relationship between Type D personality and QoL in patients with chronic diseases is well-understood, this relationship in patients with GERD has not been explored. To the best of the authors’ knowledge, this is the first study to use SEM to identify the relationships among Type D personality, symptom experience, depression and anxiety, and QoL in patients with GERD. In addition, the mediators identified in this study help elucidate promising avenues to explore in developing related interventions. Therefore, for GERD patients with a Type D personality, improved management of depression and GERD symptoms in these patients may be effective in improving their QoL.

In this study, nearly half (46.3%) of the participants were identified as having a Type D personality. This is higher than the prevalence of this personality type reported among individuals with functional gastrointestinal disorders (37.0%; Hansel et al., 2010), psoriasis (38.4%; Aguayo Carreras et al., 2020), coronary heart disease (26%; Aluja et al., 2019), and healthy individuals (20.2%; Aluja et al., 2019). Similarly, these results are higher than those reported in studies on other chronic illnesses conducted in Korea (18.4%–34.1%; Kim, Kim, et al., 2021; Kim, Nho, et al., 2021; Park et al., 2022). This finding suggests health care professionals should evaluate whether their patients with GERD have a Type D personality.

Anxiety, depression, and QoL scores were shown to be higher in the Type D personality group than in the non–Type D personality group. These results are consistent with previous studies showing Type D personality to be associated with higher anxiety and depression and with lower QoL in patients with chronic diseases (Aguayo Carreras et al, 2020; Can & Tuman, 2020; Hansel et al., 2010; Kim, Kim, et al., 2021; Kim, Nho, et al., 2021). In contrast, the results of this study indicated no significant discrepancy in GERD symptoms between Type D personality and non–Type D personality groups. This may be due to the relatively small sample size used in this study. Nevertheless, despite this lack of a statistically significant between-group difference (*p*=.076), the GERD score was higher in the Type D personality group. Moreover, having a Type D personality was found in the SEM to indirectly affect the GERD symptom experience. In other words, having a Type D personality exacerbates depression, which negatively affects GERD symptoms. This finding is similar to a previous study in which Type D personality was linked to symptom experiences (Kim et al., 2018). In addition, having a Type D personality was found in the SEM to directly affect depression and QoL. Because Type D personality can directly or indirectly affect symptoms, depression, and QoL in patients with GERD, it is necessary to evaluate and manage depression and GERD symptoms in patients with this personality type to improve their QoL.

In this study, in the SEM, GERD symptoms were found to directly affect QoL and depression was found to have direct and indirect effects on QoL in patients with GERD. These results are consistent with those of previous studies, demonstrating the negative impact of psychological factors and GERD-related symptoms on the QoL of patients with GERD (Alzahrani et al., 2023; Can & Tuman, 2020). Therefore, it is imperative to consider symptoms and depression when assessing the QoL of patients with GERD.

In addition, the multimediation model was analyzed using phantom variables to determine statistically significant paths. As a result, two of the three paths (Type D personality→ depression → QoL and Type D personality→ depression → GERD symptoms → QoL) were found to reach statistical significance, suggesting Type D personality not only directly diminishes QoL but also indirectly influences QoL by exacerbating GERD symptoms and depression. Therefore, it is necessary to consider Type D personality along with depression and GERD symptoms when assessing the QoL of patients with GERD. In particular, depression and symptom management may offer an effective approach to improving QoL in GERD patients with a Type D personality. Moreover, non–Type D individuals may also experience high depression and symptoms and lower QoL. As depression and GERD symptoms are closely related to QoL, QoL may be improved by reducing depression and GERD symptoms. Therefore, even though high depression and symptoms and lower QoL are more common in individuals with Type D personality, strategies for improving QoL may include assessing the level of depression and GERD symptoms in both the Type D personality and other personality-type groups and providing psychological interventions and symptom management to ameliorate these factors.

In this study, seven health behaviors were investigated. The Type D personality group was shown to have a higher frequency of insufficient sleep, which is consistent with findings among patients with psoriasis and myofascial pain syndrome (Aguayo Carreras et al., 2020; Can & Tuman, 2020). Although not statistically significant, a higher frequency of late-night snacking, dinner within 2 hr of going to bed, a lower frequency of breakfast, and fast eating were more frequent in the Type D personality group, potentially exacerbating their GERD symptoms (Zhang et al., 2021). The findings of previous studies indicate that individuals with Type D personality exhibit a significantly lower prevalence than their non–Type D peers of engaging in appropriate health behaviors such as following healthy dietary habits.

In addition, health behaviors such as eating habits, smoking, obesity, and exercise habits have been identified as significant contributors to the onset and progression of symptoms in patients with GERD (Wang et al., 2021; Zhang et al., 2021). Consequently, lifestyle modification, along with proton pump inhibitor medication and surgical procedures such as fundoplication, is a pivotal facet of GERD treatment (Chhabra & Ingole, 2022; Maret-Ouda et al., 2020). In future studies, the relationship between Type D personality and health behaviors, including unhealthy eating habits and insufficient sleep in patients with GERD, should be investigated and clarified.

In this study, patients with GERD with a disease duration of 3 years or less exhibited a higher QoL than their peers with a duration of >3 years. The strong correlation found between GERD duration and symptom duration aligns with the findings of previous studies, suggesting that patients with shorter symptom duration report better QoL than those with prolonged symptom duration (Lail et al., 2019). Therefore, it is imperative to actively implement interventions aimed at preventing chronicity and recurrence of GERD.

This study was affected by several limitations. First, conducting the study at a single institution may limit the generalizability of the results. Second, this study did not investigate all potentially relevant demographic or clinical factors, such as perceived socioeconomic status, treatment modalities (e.g., medication or surgery), comorbidities, and social factors (e.g., social support). Future studies should examine and incorporate these factors to explore their influence on GERD symptoms and QoL. Third, the data collection period was long because it coincided with the coronavirus disease 2019 pandemic and used a face-to-face consent and interview format. Although consistent recruitment methods and inclusion criteria were applied throughout, patients with a high fear of infectious diseases may not have visited the target hospital or may have refused to participate in the face-to-face interview. Finally, the cross-sectional study design precluded the ability to determine whether Type D personality was a cause or consequence of higher depression and anxiety, fewer health behaviors, and lower QoL. Consequently, the causal relationships identified using SEM should be interpreted as indicative rather than definitive due to the inability to establish temporal sequences or confirm true causality using a cross-sectional study design. To address these challenges, future research should implement longitudinal studies to strengthen evidence and clarify the dynamics of these associations. Despite these limitations, significant associations were identified among Type D personality, GERD experience, health behaviors, depression, anxiety, and QoL in patients with GERD. Also, causal relationships among the variables influencing QoL in patients with GERD were confirmed.

The results highlight the necessity for health care professionals to routinely assess depression, anxiety, symptoms, and QoL in patients with GERD and to assess whether patients with high levels of depression, anxiety, and symptoms and low levels of QoL have a Type D personality. In a previous study (Kim et al., 2020), lifestyle interventions based on Type D personality were shown to have positive effects such as reduced obesity, improved health-promoting behaviors, and reduced psychological distress. Another study reported on cognitive therapy in patients with coronary heart disease and Type D personality (Nejati et al., 2022). Therefore, developing cognitive therapy and lifestyle modification interventions tailored to the needs of Type D personality characteristics may prove beneficial in reducing symptoms and improving QoL in GERD patients with this personality type. Future studies are needed to evaluate the effects of interventions tailored to the Type D personality on negative emotions (including anxiety and depression), social inhibition, symptoms, and QoL in patients with GERD.

### Conclusions

In patients with GERD, having a Type D personality is significantly related to depression and anxiety severity, health behaviors, and QoL. In patients with GERD, QOL is influenced by GERD symptoms, depression, and personality type. In the SEM, GERD symptoms, depression, and having a Type D personality were all shown to affect QoL, while depression and GERD symptoms were shown to mediate the association between Type D personality and QoL.

Based on the findings, Type D personality should be assessed together with GERD symptoms and depression when assessing QoL in patients with GERD. In particular, managing depression and GERD symptoms may be beneficial strategies for enhancing QoL in GERD patients with Type D personality, and related interventions should be pursued.

## Supplementary Material
