# Supplementary material for: Relationships Among Type D Personality, Symptoms, Depression and Anxiety, and Quality of Life in Patients With Gastroesophageal Reflux Disease
Source: J Nurs Res. 2026 Jun 17;34(4):e471. doi: 10.1097/jnr.0000000000000752 (PMC13465697; doi:10.1097/jnr.0000000000000752)
Supplement: Supplementary file 1 [file jnr-34-e471-s001.docx]

**Supplementary Table 1**

Correlation between Quality of Life and Study Variables

| Variable | Age | GERD Symptoms | Depression | Anxiety | QoL_total | QoL_DA | QoL_TE | QoL_DI |
| --- | --- | --- | --- | --- | --- | --- | --- | --- |
|  | ***r*** (*p*) | | | | | | | |
| GERD symptoms | -.021 |  |  |  |  |  |  |  |
|  | (.802) |  |  |  |  |  |  |  |
| Depression | -.006 | .408 |  |  |  |  |  |  |
|  | (.947) | (< .001) |  |  |  |  |  |  |
| Anxiety | -.005 | .444 | .741 |  |  |  |  |  |
|  | (.956) | (< .001) | (< .001) |  |  |  |  |  |
| QoL_Total | .039 | -.545 | -.525 | -.462 |  |  |  |  |
|  | (.641) | (< .001) | (< .001) | (< .001) |  |  |  |  |
| QoL_DA | .132 | -.619 | -.545 | -.490 | .862 |  |  |  |
|  | (.111) | (< .001) | (< .001) | (< .001) | (< .001) |  |  |  |
| QoL_TE | .059 | -.366 | -.465 | -.374 | .865 | .672 |  |  |
|  | (.480) | (< .001) | (< .001) | (< .001) | (< .001) | (< .001) |  |  |
| QoL_DI | .086 | -.476 | -.417 | -.382 | .876 | .789 | .631 |  |
|  | (.302) | (< .001) | (< .001) | (< .001) | (< .001) | (< .001) | (< .001) |  |
| QoL_PW | -.100 | -.445 | -.415 | -.372 | .851 | .589 | .693 | .613 |
|  | (.230) | (< .001) | (< .001) | (< .001) | (< .001) | (< .001) | (< .001) | (< .001) |

*Note.* GERD = gastroesophageal reflux disease; QOL = quality of life; DA = daily activity; TE = treatment effect; DI = diet; PW = psychological well- being.
